# *catena*-Poly[[iodidocopper(I)]-μ-4,4′,6,6′-tetra­methyl-2,2′-(ethyl­enedithio)dipyrimidine-κ^2^ *N*:*N*′]

**DOI:** 10.1107/S1600536809032437

**Published:** 2009-08-22

**Authors:** Hong Bo Lu, Lin Li, Guo Qiang Lv, Jia Xiang Yang

**Affiliations:** a Key Laboratory of Special Display Technology, Hefei University of Technology, Ministry of Education, Hefei 230009, People’s Republic of China; b Academy of Opto-Electronic Technology, Hefei University of Technology, Ministry of Education, Hefei 230009, People’s Republic of China; c Department of Chemistry, Anhui University, Hefei 230039, People’s Republic of China

## Abstract

In the title coordination polymer, [CuI(C_14_H_18_N_4_S_2_)]_*n*_, the Cu^I^ center is trigonally coordinated by two pyrimidine N-atom donors from two distinct dithio­ether ligands and one iodide anion. The Cu and I atoms are located on a twofold axis, whereas the midpoint of the central C—C bond of the dithio­ether ligand is located on an inversion center. Each organic ligand, acting in a bidentate mode, bridges two Cu^I ^ions, resulting in the formation of polymeric zigzag chains. The dihedral angle between the two pyrimidine units bonded to the metal center is 88.01 (2)°. The crystal packing is mainly stabilized by van der Waals forces and π–π stacking inter­actions, with an inter­planar distance between the pyrimidine rings of adjacent chains of 3.638 (3) Å.

## Related literature

For applications of closed-shell metal atoms or ions, see: Catalano *et al.* (2000 ▶). For applications of conjugated multi-branched mol­ecules in optical materials, see: Nishihara *et al.* (1989 ▶); Roberto *et al.* (2000 ▶). For the structures of CuI complexes with similar ligands, see: Shi *et al.* (2008 ▶).
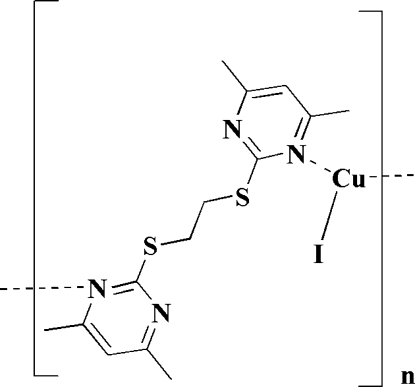


## Experimental

### 

#### Crystal data

[CuI(C_14_H_18_N_4_S_2_)]
*M* *_r_* = 496.88
Monoclinic, 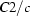

*a* = 14.201 (5) Å
*b* = 8.064 (5) Å
*c* = 16.940 (5) Å
β = 111.655 (5)°
*V* = 1803.0 (14) Å^3^
*Z* = 4
Mo *K*α radiation
μ = 3.16 mm^−1^
*T* = 293 K
0.33 × 0.24 × 0.21 mm

#### Data collection

Bruker APEXII CCD area-detector diffractometer
Absorption correction: multi-scan (*SADABS*; Sheldrick, 1996 ▶) *T*_min_ = 0.419, *T*_max_ = 0.515
5585 measured reflections
2070 independent reflections
1942 reflections with *I* > 2σ(*I*)
*R*_int_ = 0.012

#### Refinement

*R*[*F* ^2^ > 2σ(*F* ^2^)] = 0.019
*wR*(*F* ^2^) = 0.053
*S* = 1.09
2070 reflections
103 parameters
H-atom parameters constrained
Δρ_max_ = 0.51 e Å^−3^
Δρ_min_ = −0.37 e Å^−3^

### 

Data collection: *APEX2* (Bruker, 2005 ▶); cell refinement: *SAINT* (Bruker, 2005 ▶); data reduction: *SAINT*; program(s) used to solve structure: *SIR97* (Altomare *et al.*, 1999 ▶); program(s) used to refine structure: *SHELXL97* (Sheldrick, 2008 ▶); molecular graphics: *SHELXTL* (Sheldrick, 2008 ▶); software used to prepare material for publication: *WinGX* (Farrugia, 1999 ▶).

## Supplementary Material

Crystal structure: contains datablocks I, global. DOI: 10.1107/S1600536809032437/gk2225sup1.cif

Structure factors: contains datablocks I. DOI: 10.1107/S1600536809032437/gk2225Isup2.hkl

Additional supplementary materials:  crystallographic information; 3D view; checkCIF report

## Figures and Tables

**Table:** 

I1—Cu1	2.5191 (16)
Cu1—N2	2.0327 (16)

**Table:** 

N2^i^—Cu1—N2	118.55 (10)
N2^i^—Cu1—I1	120.72 (5)

Symmetry code: (i) 
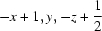
.

**Fig. 1.**
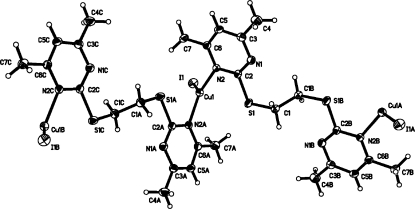
: The molecular structure of the title compound showing 30% probability displacement ellipsoids. Atoms labelled with the suffixes A, B and C are at the symmetry positions (1 - x, y, 0.5 - z), (1 - x, 1 - y, -z) and (x, 1 - y, -1/2 + z), respectively.

## supplementary crystallographic information

### Comment

Previous studies have shown that the bonding interaction between closed-shell metal atoms or ions is gaining increasing attention (Catalano *et al.*, 2000), there are a few reports of similar association in the case of alkyl copper (I) complexes. Heterocycle-based aromatic systems with conjugated multi-branched structure possess potential applications in optical image processing, all-optical switching, and integrated optical devices (Nishihara *et al.*, 1989; Roberto *et al.*, 2000). Pyrimidine is a π-electron deficient with its ionization potential value of 10.41 eV and metal complexes of such ligand has been reported (Shi *et al.*, 2008). On the other hand, pyrimidine ring has well known reactivity in the positions 4 and 6, which can easily undergo reactions with an aromatic aldehyde in solvent-free condition. Therefore we pay our attention to the pyrimidine system. As part of our ongoing investigation on d^10^ ions and pyrimidine derivatives, the title compound, has been prepared and its crystal structure is presented here.

The molecular structure of the title compound shows that Cu atom coordinated in a triangle-planar configuration (Fig. 1) with two equal Cu—N and one Cu—I bonds (Table 1). The dihedral angles formed by the two pyrimidine rings (N1, C2, N2, C6, C5, C3 and N1A, C2A, N2A, C6A, C5A, C3A) is 88.01 (2)°. Each ligand, acting in a bidentate mode, bridges two Cu ions, resulting in the formation of polymeric zigzag chains. The crystal packing is mainly stabilized by van der Waals forces and π-π interactions, with the shortest distance of 3.938 (3)Å along *c* axis.

### Experimental

A mixture of 4,4',6,6'-tetramethyl-2,2'-(ethylenedithio)dipyrimidine (0.30 mmol) and CuI (0.30 mmol) was heated at 363 K with CHCl_3_ (20 ml) as a solvent for 10 h. The red powder of the title compound was filtered and washed thoroughly with water and then air dried (yield 55%). Single crystals suitable for X-ray analysis were obtained by slow evaporation from a dichloromethane/2-propanol (3:1) solution.

### Refinement

All H atoms were positioned geometrically with C—H =0.97 and 0.96 Å for methylene and methyl H atoms, respectively, and constrained to ride on their parent atoms, with *U*_iso_(H) = *xU*_eq_(C), where *x* = 1.5 for methyl H and *x* = 1.2 for methylene H atoms.

### Crystal data

**Table:**

[CuI(C_14_H_18_N_4_S_2_)]	*F*(000) = 976
*M**_r_* = 496.88	*D*_x_ = 1.830 Mg m^−^^3^
Monoclinic, *C*2/*c*	Mo *K*α radiation, λ = 0.71069 Å
Hall symbol: -C 2yc	Cell parameters from 4275 reflections
*a* = 14.201 (5) Å	θ = 3.0–27.5°
*b* = 8.064 (5) Å	µ = 3.16 mm^−^^1^
*c* = 16.940 (5) Å	*T* = 293 K
β = 111.655 (5)°	Prism, yellow
*V* = 1803.0 (14) Å^3^	0.33 × 0.24 × 0.21 mm
*Z* = 4	

### Data collection

**Table:**

Bruker APEXII CCD area-detector diffractometer	2070 independent reflections
Radiation source: sealed tube	1942 reflections with *I* > 2σ(*I*)
graphite	*R*_int_ = 0.012
Detector resolution: 0 pixels mm^-1^	θ_max_ = 27.5°, θ_min_ = 3.0°
φ and ω scans	*h* = −18→14
Absorption correction: multi-scan (*SADABS*; Sheldrick, 1996)	*k* = −10→9
*T*_min_ = 0.419, *T*_max_ = 0.515	*l* = −21→21
5585 measured reflections	

### Refinement

**Table:**

Refinement on *F*^2^	Primary atom site location: structure-invariant direct methods
Least-squares matrix: full	Secondary atom site location: difference Fourier map
*R*[*F*^2^ > 2σ(*F*^2^)] = 0.019	Hydrogen site location: inferred from neighbouring sites
*wR*(*F*^2^) = 0.053	H-atom parameters constrained
*S* = 1.09	*w* = 1/[σ^2^(*F*_o_^2^) + (0.0273*P*)^2^ + 1.5073*P*] where *P* = (*F*_o_^2^ + 2*F*_c_^2^)/3
2070 reflections	(Δ/σ)_max_ = 0.001
103 parameters	Δρ_max_ = 0.51 e Å^−^^3^
0 restraints	Δρ_min_ = −0.37 e Å^−^^3^

### Special details

**Table:**

Geometry. All e.s.d.'s (except the e.s.d. in the dihedral angle between two l.s. planes) are estimated using the full covariance matrix. The cell e.s.d.'s are taken into account individually in the estimation of e.s.d.'s in distances, angles and torsion angles; correlations between e.s.d.'s in cell parameters are only used when they are defined by crystal symmetry. An approximate (isotropic) treatment of cell e.s.d.'s is used for estimating e.s.d.'s involving l.s. planes.
Refinement. Refinement of *F*^2^ against ALL reflections. The weighted *R*-factor *wR* and goodness of fit *S* are based on *F*^2^, conventional *R*-factors *R* are based on *F*, with *F* set to zero for negative *F*^2^. The threshold expression of *F*^2^ > σ(*F*^2^) is used only for calculating *R*-factors(gt) *etc*. and is not relevant to the choice of reflections for refinement. *R*-factors based on *F*^2^ are statistically about twice as large as those based on *F*, and *R*- factors based on ALL data will be even larger.

### Fractional atomic coordinates and isotropic or equivalent isotropic displacement parameters (Å^2^)

**Table:**

	*x*	*y*	*z*	*U*_iso_*/*U*_eq_	
I1	0.5000	−0.19054 (3)	0.2500	0.04737 (8)	
Cu1	0.5000	0.12185 (4)	0.2500	0.03416 (9)	
S1	0.52076 (4)	0.34963 (8)	0.11434 (3)	0.04371 (14)	
N1	0.32885 (12)	0.4555 (2)	0.05807 (11)	0.0342 (3)	
N2	0.38109 (11)	0.2506 (2)	0.16787 (9)	0.0290 (3)	
C5	0.21161 (14)	0.3484 (3)	0.11508 (12)	0.0344 (4)	
H5	0.1466	0.3468	0.1161	0.041*	
C3	0.23460 (14)	0.4530 (2)	0.06006 (12)	0.0340 (4)	
C6	0.28616 (14)	0.2463 (3)	0.16853 (11)	0.0317 (4)	
C1	0.52381 (15)	0.5214 (3)	0.04659 (12)	0.0366 (4)	
H1A	0.4881	0.6149	0.0584	0.044*	
H1B	0.5936	0.5545	0.0597	0.044*	
C2	0.39619 (13)	0.3559 (2)	0.11190 (11)	0.0298 (3)	
C4	0.15807 (18)	0.5669 (3)	0.00052 (16)	0.0505 (5)	
H4A	0.1729	0.6791	0.0202	0.076*	
H4B	0.0916	0.5374	−0.0017	0.076*	
H4C	0.1605	0.5575	−0.0552	0.076*	
C7	0.26594 (15)	0.1282 (3)	0.22824 (14)	0.0437 (5)	
H7A	0.2754	0.0165	0.2128	0.066*	
H7B	0.1975	0.1419	0.2252	0.066*	
H7C	0.3119	0.1500	0.2851	0.066*	

### Atomic displacement parameters (Å^2^)

**Table:**

	*U*^11^	*U*^22^	*U*^33^	*U*^12^	*U*^13^	*U*^23^
I1	0.04987 (13)	0.03860 (12)	0.05587 (13)	0.000	0.02211 (9)	0.000
Cu1	0.03150 (16)	0.0412 (2)	0.03022 (16)	0.000	0.01191 (12)	0.000
S1	0.0306 (2)	0.0610 (3)	0.0444 (3)	0.0086 (2)	0.0194 (2)	0.0221 (2)
N1	0.0331 (8)	0.0374 (9)	0.0338 (8)	0.0040 (6)	0.0144 (6)	0.0047 (6)
N2	0.0270 (7)	0.0335 (7)	0.0272 (7)	−0.0021 (6)	0.0107 (5)	0.0004 (6)
C5	0.0261 (8)	0.0388 (10)	0.0398 (10)	−0.0015 (7)	0.0138 (7)	−0.0044 (8)
C3	0.0315 (9)	0.0344 (10)	0.0346 (9)	0.0022 (7)	0.0106 (7)	−0.0033 (7)
C6	0.0305 (8)	0.0352 (9)	0.0308 (8)	−0.0052 (7)	0.0130 (7)	−0.0036 (7)
C1	0.0331 (9)	0.0444 (11)	0.0337 (10)	−0.0086 (8)	0.0141 (8)	0.0027 (8)
C2	0.0292 (8)	0.0343 (9)	0.0283 (8)	−0.0006 (7)	0.0133 (7)	−0.0001 (7)
C4	0.0399 (11)	0.0518 (13)	0.0563 (13)	0.0140 (10)	0.0136 (10)	0.0122 (11)
C7	0.0338 (10)	0.0531 (13)	0.0470 (11)	−0.0064 (9)	0.0183 (8)	0.0099 (10)

### Geometric parameters (Å, °)

**Table:**

I1—Cu1	2.5191 (16)	C3—C4	1.495 (3)
Cu1—N2^i^	2.0327 (16)	C6—C7	1.492 (3)
Cu1—N2	2.0327 (16)	C1—C1^ii^	1.510 (4)
S1—C2	1.7550 (19)	C1—H1A	0.9700
S1—C1	1.809 (2)	C1—H1B	0.9700
N1—C2	1.322 (2)	C4—H4A	0.9600
N1—C3	1.351 (2)	C4—H4B	0.9600
N2—C2	1.348 (2)	C4—H4C	0.9600
N2—C6	1.353 (2)	C7—H7A	0.9600
C5—C3	1.382 (3)	C7—H7B	0.9600
C5—C6	1.383 (3)	C7—H7C	0.9600
C5—H5	0.9300		
			
N2^i^—Cu1—N2	118.55 (10)	S1—C1—H1A	109.1
N2^i^—Cu1—I1	120.72 (5)	C1^ii^—C1—H1B	109.1
N2—Cu1—I1	120.72 (5)	S1—C1—H1B	109.1
C2—S1—C1	102.87 (9)	H1A—C1—H1B	107.8
C2—N1—C3	116.41 (16)	N1—C2—N2	127.28 (16)
C2—N2—C6	116.10 (16)	N1—C2—S1	120.02 (13)
C2—N2—Cu1	119.73 (12)	N2—C2—S1	112.69 (13)
C6—N2—Cu1	124.04 (13)	C3—C4—H4A	109.5
C3—C5—C6	119.38 (17)	C3—C4—H4B	109.5
C3—C5—H5	120.3	H4A—C4—H4B	109.5
C6—C5—H5	120.3	C3—C4—H4C	109.5
N1—C3—C5	120.57 (17)	H4A—C4—H4C	109.5
N1—C3—C4	117.01 (18)	H4B—C4—H4C	109.5
C5—C3—C4	122.42 (18)	C6—C7—H7A	109.5
N2—C6—C5	120.24 (17)	C6—C7—H7B	109.5
N2—C6—C7	117.63 (17)	H7A—C7—H7B	109.5
C5—C6—C7	122.13 (17)	C6—C7—H7C	109.5
C1^ii^—C1—S1	112.45 (19)	H7A—C7—H7C	109.5
C1^ii^—C1—H1A	109.1	H7B—C7—H7C	109.5
			
N2^i^—Cu1—N2—C2	59.68 (13)	C3—C5—C6—N2	1.1 (3)
I1—Cu1—N2—C2	−120.32 (13)	C3—C5—C6—C7	−178.78 (19)
N2^i^—Cu1—N2—C6	−115.97 (16)	C2—S1—C1—C1^ii^	−79.7 (2)
I1—Cu1—N2—C6	64.03 (16)	C3—N1—C2—N2	0.9 (3)
C2—N1—C3—C5	−1.1 (3)	C3—N1—C2—S1	179.92 (14)
C2—N1—C3—C4	179.03 (19)	C6—N2—C2—N1	0.3 (3)
C6—C5—C3—N1	0.2 (3)	Cu1—N2—C2—N1	−175.69 (15)
C6—C5—C3—C4	−179.9 (2)	C6—N2—C2—S1	−178.79 (13)
C2—N2—C6—C5	−1.3 (3)	Cu1—N2—C2—S1	5.22 (18)
Cu1—N2—C6—C5	174.53 (14)	C1—S1—C2—N1	9.16 (18)
C2—N2—C6—C7	178.58 (18)	C1—S1—C2—N2	−171.68 (14)
Cu1—N2—C6—C7	−5.6 (2)		

Symmetry codes: (i) −*x*+1, *y*, −*z*+1/2; (ii) −*x*+1, −*y*+1, −*z*.
